# Intercellular contact and cargo transfer between Müller glia and to microglia precede apoptotic cell clearance in the developing retina

**DOI:** 10.1242/dev.202407

**Published:** 2024-01-04

**Authors:** Michael Morales, Anna P. Findley, Diana M. Mitchell

**Affiliations:** Department of Biological Sciences, University of Idaho, Moscow, ID 83844, USA

**Keywords:** Müller glia, Phagocytosis, Microglia, Intercellular transfer, Time-lapse recordings, Apoptosis, Zebrafish

## Abstract

To clarify our understanding of glial phagocytosis in retinal development, we used real-time imaging of larval zebrafish to provide cell-type specific resolution of this process. We show that radial Müller glia frequently participate in microglial phagocytosis while also completing a subset of phagocytic events. Müller glia actively engage with dying cells through initial target cell contact and phagocytic cup formation, after which an exchange of the dying cell from Müller glia to microglia often takes place. In addition, we find evidence that Müller glia cellular material, possibly from the initial Müller cell phagocytic cup, is internalized into microglial compartments. Previously undescribed Müller cell behaviors were seen, including cargo splitting, wrestling for targets and lateral passing of cargo to neighbors. Collectively, our work provides new insight into glial functions and intercellular interactions, which will allow future work to understand these behaviors on a molecular level.

## Teaser

**Summary:** Real-time imaging of developing zebrafish retinas reveals intercellular exchanges between Müller glial cells and with microglia during the clearance of apoptotic cells.

## INTRODUCTION

Cell death occurs in tissues and organs during development and throughout the life of an animal and these dying cells require clearance by other cells through phagocytosis. The process of phagocytosis has been well-studied in a variety of cells and tissues, and in vertebrates there is a class of professional phagocytes known as macrophages which, as tissue residents, efficiently perform this function across the body (Gordon and Plüddemann, 2017). Within the central nervous system, the specialized population of resident macrophages known as microglia perform phagocytosis, as well as additional specialized functions, in this very complex and delicate neural tissue. Recently, the function of microglia has risen to greater interest in the contexts of homeostasis, pathology and regeneration. The phagocytic function of microglia is indeed well-appreciated, yet also in the central nervous system exist neuroglial cells that have become known to engage in phagocytic activity (Jung and Chung, 2018). Within the retina in particular are the Müller glia (MG), which have been reported to engage in phagocytosis in a variety of species and contexts (Bailey et al., 2010; Lew et al., 2022; Nomura-Komoike et al., 2020; Sakami et al., 2019; Thiel et al., 2022b). Despite this body of work, the extent of such phagocytic activity is not yet fully defined and has not been directly observed *in vivo* in real-time.

The retina is the neural component of the eye and serves to convert incoming visual stimuli into the experience of vision through the use of specialized neurons. MG are neuroglial cells that extend radially across the retina, spanning the full breadth of the neuronal components of the retina (Güngör Kobat and Turgut, 2020). Müller cells are known to crucially support the neurons that they neighbor by providing various homeostatic functions such as metabolite and neurotransmitter synthesis and recycling (Pfeiffer et al., 2020). Müller cells have also garnered a special interest due to their ability to produce neuronal progenitors leading to regeneration of the retina following damage in zebrafish (Bernardos et al., 2007; Fausett and Goldman, 2006; Nagashima et al., 2013; Thummel et al., 2008). Further, MG-microglia interactions appear to influence such regenerative potential (Fischer et al., 2014; Fogerty et al., 2022; Todd et al., 2020; White et al., 2017). Phagocytosis, or engulfment of dying neurons by retinal MG, has been described in several studies through static visualization of dying cell markers within Müller cells (Bailey et al., 2010; Egensperger et al., 1996; Krylov et al., 2023; Lew et al., 2022; Morris et al., 2005; Nomura-Komoike et al., 2020; Sakami et al., 2019; Thiel et al., 2022b). In some cases, these reports appear to contrast with evidence that microglia dominate clearance of dying cells during retinal development (Blume et al., 2020; Francisco-Morcillo et al., 2014; Thiel et al., 2022b) or following induced retinal damage (Mitchell et al., 2018; White et al., 2017). Our recent work demonstrated the ability of MG to substantially increase phagocytosis in the absence of microglia in an otherwise normally developing retina (Thiel et al., 2022b). However, to our knowledge to date, studies reporting MG phagocytosis *in vivo* involve primarily fixed tissue or static samples and the full process of Müller glial phagocytosis *in vivo*, in real-time, has not been documented.

When differentiated, Müller glial bodies are stationary within the inner nuclear layer of the retina. Because Müller glial processes enwrap neurons, there remains the possibility that engulfment occurs as Müller processes collapse around already enwrapped neurons as they die. Alternatively, MG may actively sense and reach dying cells with cellular processes that then enclose around the target. In addition, given that we and others reported that microglia are the dominant phagocyte in the developing CNS (Blume et al., 2020; Casano et al., 2016; Mazaheri et al., 2014; Möller et al., 2022), there remains a strong possibility that interactions between microglia and other cell types such as the MG occur during, and to facilitate, the clearance process. While early endosomal markers support active engulfment of dying cells by MG in the absence of microglia (Thiel et al., 2022b), lysosomal fusion of such compartments upon engulfment within MG has not been clearly demonstrated. In summary, static images do not have the power to sufficiently reveal MG phagocytic propensity or to visualize transient interactions of MG with microglia and/or dying cells.

We used time-lapse *in vivo* imaging of transgenic zebrafish reporter lines to address these outstanding questions by simultaneously recording MG, microglia and dying cells in the developing retina. Our real-time observations reconcile seemingly contradictory reports of MG versus microglia phagocytosis of dying cells, suggest that clearance of dying cells in the vertebrate retina involves both MG and microglia, and reveal previously undocumented, remarkably dynamic behaviors and intercellular interactions of MG with microglia and their neighbors that should initiate an evolution of our view of how these glial cells function.

## RESULTS

### MG actively and dynamically extend processes towards dying cells

To visualize MG, microglia and dying cells simultaneously during retinal development, we performed time-lapse imaging of triple transgenic larval zebrafish expressing fluorescent protein reporters for MG (*TP1:mTurquoise*), microglia (*mpeg1:mCherry*; Ellett et al., 2011) and apoptotic cells (*TBP:Gal4; UAS:SecA5-YFP*, henceforth referred to as ‘SecA5-YFP’; Blume et al., 2020; van Ham et al., 2010). The *TP1:mTurquoise* line reports Notch signaling via the TP1 transcriptional element, which labels MG after ∼48 h post fertilization (hpf) (MacDonald et al., 2015). The SecA5-YFP reporter marks exposed phosphatidyl serine (PtdSer) on the surface of presumptively apoptotic cells via YFP fused to AnnexinA5 (AnnA5). Exposure of PtdSer is an early apoptotic event (Martin et al., 1995), thus the SecA5-YFP reporter allows visualization of apoptotic cell bodies via YFP (Herzog et al., 2019; Mazaheri et al., 2014; van Ham et al., 2010, 2012). Zebrafish microglia are visualized using *mpeg1* (also known as *mpeg1.1*) driven transgenes (Blume et al., 2020; Ellett et al., 2011; Mitchell et al., 2018). Zebrafish larvae were imaged beginning at an age of ∼2.5 days post fertilization (dpf) or ∼58 hpf for a total duration of 10 h, at 3-min intervals, coincidental with a wave of apoptosis in the retina previously described (Biehlmaier et al., 2001), and which we have previously visualized with the *mpeg1* and SecA5-YFP reporters (Blume et al., 2020).

**Fig. 1.**
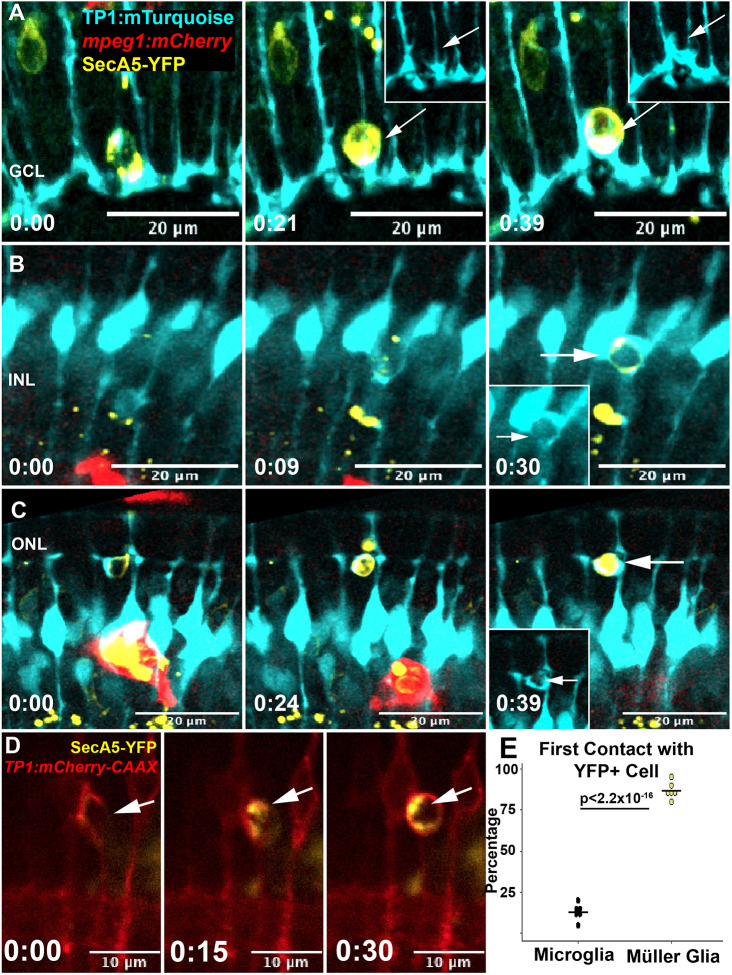
**Müller cell process extension and contact with dying cells.** (A) Time-lapse stills show a Müller glial cell (*TP1:mTurquoise*) recognizing a dying cell (SecA5-YFP^+^) in the ganglion cell layer (GCL) and extending a process towards and around the emerging YFP^+^ cell. Insets at 0:21 and 0:39 (h:min) show the Müller cell forming a phagocytic cup-like structure around the YFP^+^ cell (arrow). (B) Time-lapse stills show Müller glia (MG) process extension around a YFP^+^ cell in the inner nuclear layer (INL). Inset at 0:30 shows phagocytic cup structure (arrow). (C) Time-lapse stills show an extension of MG processes around a YFP^+^ cell in the outer retina (outer nuclear layer; ONL). Inset at 0:39 shows the phagocytic cup structure (arrow). (D) Membrane reporter expressed by MG (*TP1:mCherry*-CAAX) further demonstrates the extension of processes around a SecA5-YFP^+^ dying cell forming a phagocytic cup, often enwrapping the YFP^+^ cell (arrows). In this example, the extension of the mCherry^+^ cellular processes begins before (time stamp 0:00) the appearance of the YFP signal (time stamp 0:15). Time stamps in all panels, bottom left (h:min), are relative to the first frame in the time series. (E) Quantification of cell type initially contacting a YFP^+^ cell. Percentage was calculated using the number of YFP^+^ cells initially contacted by microglia or MG out of the total YFP^+^ cells that appeared and were cleared during the recording session. Each data point represents the percentage of the YFP^+^ cells first contacted by microglia or MG in each of the separate recordings. *P*-value (binomial proportion test) is shown.

Consistent with our previous study (Blume et al., 2020), microglia were seen to engulf YFP^+^ cell bodies throughout the retina (Movie 1). In addition, now observable due to the MG reporter, we observed that MG were active and dynamic, extending processes from the MG cell body towards SecA5-YFP^+^ targets in their vicinity (Movies 1 and 2; Fig. 1). These processes extended around the body of a YFP^+^ cell, often forming a compartment-like structure resembling a phagocytic cup (Fig. 1A-C). We observed the contact of MG processes with dying cells in both the apical, inner and basal regions of the retina, suggesting that the MG respond to death of numerous types of neurons and in all regions of the retina (Fig. 1A-C). Consistent with death of inner retinal cells during this time window (Biehlmaier et al., 2001), we observed cell death arising in the inner retina. Notably, however, we did not observe SecA5-YFP^+^ MG in any of our recordings (see Table S1), nor did we observe compaction or fragmentation of any MG processes or cell bodies. Consistent with these observations, our previous work did not detect significant numbers of cleaved caspase 3^+^ MG at a corresponding age (Thiel et al., 2022b), indicating that MG themselves do not undergo a wave of cell death during this timeframe.

Because the *TP1:mTurquoise* transgene encodes a cytoplasmic reporter expressed in MG, we wanted to visualize a fuller extent of this behavior using a membrane-tagged reporter. To do this, we used the reporter line *TP1:mCherry-CAAX* (Thiel et al., 2022b), in which MG express a membrane-tagged mCherry reporter coupled with the SecA5-YFP reporter. We again visualized Müller cell processes extending towards and around the YFP^+^ dying cell (Fig. 1D). Interestingly, process extension from the Müller cell often initiated before detection of the YFP^+^ cell death reporter (Fig. 1D), suggesting that the MG respond to signals released from the dying cell as cell death is executed.

Given the observed response of MG to dying cells, and the known role of microglia in clearance during this time (Blume et al., 2020), we quantified clearance of SecA5-YFP^+^ cells in six unique recordings from six different larval *TP1:mTurquiose*;SecA5-YFP;*mpeg1:mCherry* triple transgenic zebrafish. For such quantifications, we selected YFP^+^ cells that were visually detected then terminally cleared within the duration of the 10-h recording. The total numbers of YFP^+^ cells tracked for such analyses, and the cell type seen to engage with these YFP^+^ cells, are provided in Table S1. To determine which cell type most frequently contacted a YFP^+^ cell, we calculated the percent of initial contacts by cell type in each of our recordings (Fig. 1E). We found that the majority of SecA5-YFP^+^ cells detected and cleared during this period are initially contacted by MG via the extension of processes from the Müller cell (Fig. 1E) rather than microglia, revealing that MG are remarkably active in sensing and contacting dying cells.

### Apoptotic cargo and Müller glial cellular material is transferred from MG to microglia

**Fig. 2.**
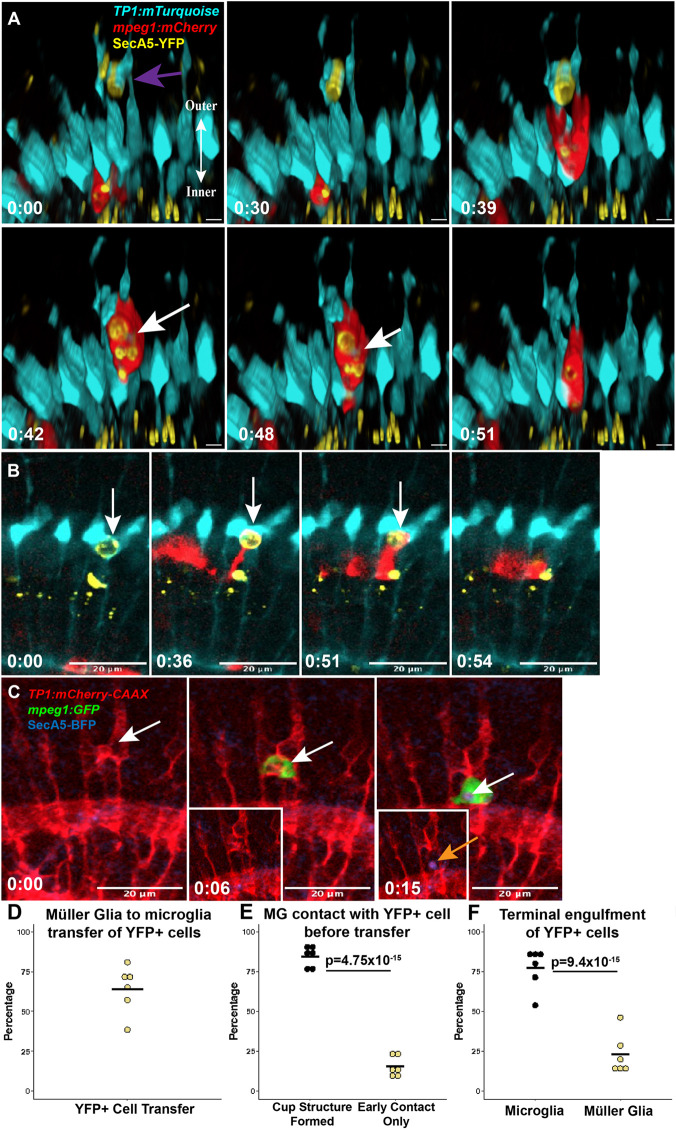
**Transfer of cargo and cellular material from Müller glia to microglia.** (A) Time-lapse stills (3D rendered) showing a Müller cell (cyan) engaging a YFP^+^ dying cell (purple arrow) in the apical retina and enwrapping the target with cellular processes and a cup structure before a microglial cell (red) emerges and retrieves the cargo. Pieces of the Müller cell cytoplasmic reporter are detected and move with the YFP^+^ cargo (white arrow), suggesting cytoplasmic material transfer. See Movie 3. Scale bar: ∼8 µm. (B) Time-lapse stills show an inner retinal YFP^+^ cell initially engaged by Müller glia (MG; arrow), with a phagocytic cup-like structure formed around the cell before the YFP^+^ cell is transferred to microglia (red). See Movie 4. (C) Alternative transgenic reporter combination to label MG via membrane-localized mCherry (red), microglia with GFP (green) and apoptotic cells with BFP (blue). Time-lapse stills show a microglial cell (green) interacting with an mCherry-CAAX^+^ (red) MG compartment (white arrow). The microglial cell retrieves the cargo from the Müller cell, taking the mCherry membrane reporter (red) co-labeled with SecA5-BFP (orange arrow, inset, 0:15). Time stamps in panels A and B, bottom left (h:min), are relative to the first frame in the time series. (D) Quantification of events in which YFP^+^ cargo originally engaged by MG was then commandeered by microglia, for each recording session. Percentages were determined by the number of YFP^+^ cells that were transferred to microglia out of the number of YFP^+^ cells initially contacted by MG. (E) For YFP^+^ cargo transfer events (MG to microglia), the first contact of YFP^+^ cells by MG were scored as either cup structure formed or early contact only. The relative percentages of these contact types by MG are shown for each recording. *P*-value (binomial proportion test) is shown. (F) The percentage of SecA5-YFP^+^ cells terminally engulfed by microglia or MG out of the total number of cleared YFP^+^ cells. Each data point represents the percentage calculated for each recording. *P*-value (binomial proportion test) is shown.

Our previous studies have demonstrated that microglia dominate dying cell clearance in the developing zebrafish retina (Blume et al., 2020; Thiel et al., 2022b). Visualizing MG in addition to microglia in real-time revealed that, although MG primarily make the first contact (Fig. 1), the process of dying cell engulfment often involved interaction of both MG and microglia with the dying cells. In particular, we observed that SecA5-YFP^+^ cells initially engaged and enveloped by Müller cell processes are frequently commandeered by microglia (Movies 3 and 4; Fig. 2). In a general synopsis of this sequence, the onset of YFP signal is met with contact by Müller cell processes forming a phagocytic cup-like structure that envelops the YFP^+^ target. During this process, migratory microglia are presumably in transit to the site of the YFP^+^ cell and, upon arrival, the microglial cell executes a maneuver by which the YFP^+^ cell enveloped by the Müller cell processes is transferred to the microglia cell for terminal engulfment (Fig. 2A,B; Movies 3 and 4).

**Fig. 3.**
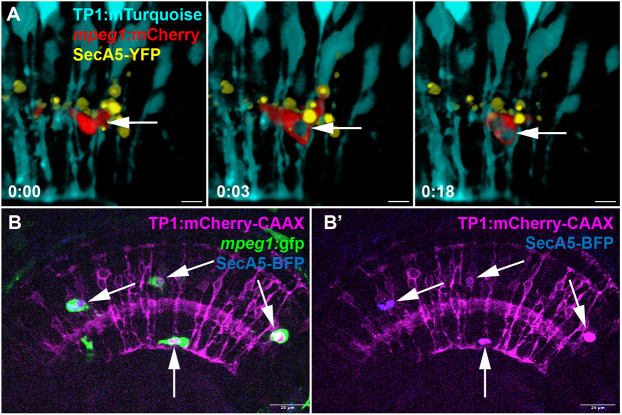
**Detection of Müller glia components inside microglial compartments.** (A) In our recordings using *TP1:mTurquoise* as the Müller cell reporter, we also observed, at times, Müller cell cytoplasmic reporter inside of microglial compartments (arrow). Time stamps, bottom left (h:min), relative to the first frame in the time series. See Movie 5. (B,B′) Using the membrane-localized reporter *TP1:mCherry*-CAAX, expressed by Müller glia, we observe the mCherry MG reporter inside of microglial compartments (arrows). This occasionally, but not always, co-localized with the SecA5-BFP reporter. The mCherry-CAAX signal is retained as microglia migrate through the retina; see Movie 6. Scale bars: 20 µm.

Our quantifications show that the transfer of YFP^+^ cells initially contacted/enwrapped by Müller cell processes to microglia occurred frequently for the YFP^+^ cells analyzed (Fig. 2D). The majority of these YFP^+^ cell cargo transfers occurred after a cup-like structure had been formed by MG, though a smaller proportion were transferred to microglia after having received only early contact by Müller cell processes (Fig. 2E). Ultimately, terminal engulfment of YFP^+^ cells by microglia occurred more frequently than by MG (Fig. 2F), despite the initial contact being more frequently detected by MG (Fig. 1E). As we observed this process of YFP^+^ engulfment and transfer, we also observed mTurquoise^+^ signal, representing the Müller cell cytoplasmic reporter, transferred to the microglia along with the YFP^+^ cargo (Fig. 2A, white arrow). In other recordings, we again detected mTurquoise^+^ MG cytoplasmic reporter inside of microglial compartments (Movie 5; Fig. 3A). Interestingly, this indicates that, in addition to transfer of the enwrapped dying cells from MG to microglia, some of the Müller cell cytoplasm is also transferred.

To further visualize this interaction and cargo transfer, we used an alternative set of transgenic reporters in which the MG were visualized by membrane-tagged mCherry (*TP1:mCherry-CAAX*), microglia with *mpeg1:gfp* (Ellett et al., 2011) and dying cells with *bact2:secA5-mTagBFP* (hereafter SecA5-BFP). In this triple transgenic system, the membrane-tagged reporter demonstrated the formation of Müller cell process-derived compartments, which preceded the engagement by microglia and the BFP signal (Fig. 2C). The primary purpose of this reporter combination was to support our previous observations with a different reporter system. Microglia engaged with Müller cell compartments, with a concurrent detection of the BFP^+^ cell death reporter (Fig. 2C, orange arrow). However, unlike the YFP reporter for PtdSer-exposing cells, the BFP reporter did not reliably report before compartment formation and the signal overall was weak, though it became more prominent once inside of microglia. We consider that differences in visualizing PtdSer-exposing cells with the YFP versus BFP reporters is a result of inherent differences in transgene drivers/expression, fluorophore brightness, as well as acid sensitivity (Subach et al., 2008; Young et al., 2010). Transfer of dying cell cargo from MG to microglia was again supported as the onset of BFP signal occurred upon interaction of microglia with the MG compartment, and the BFP signal then traveled with the microglial cell. Interestingly, what was most apparent from the combination of these reporters was the detection of MG membrane-tagged mCherry signal within microglia compartments (Movie 6; Fig. 3B). Strong mCherry^+^ membrane signal is persistently visible within the migratory microglia (Fig. 2C, time 0:15, orange arrow). As stated above for the SecA5-YFP reporter, we again did not observe BFP^+^ MG or collapse of MG, indicating that the engulfment of MG is unlikely to be a significant origin of the Müller cell reporter signals (cytoplasmic or membrane tagged) detected within microglia. Rather, the observations from both the MG cytoplasmic and membrane-tagged reporters suggest that cellular material is transferred from MG to microglia concomitant with that of dying cell cargo.

### MG complete engulfment of apoptotic cells in a subset of clearance events

**Fig. 4.**
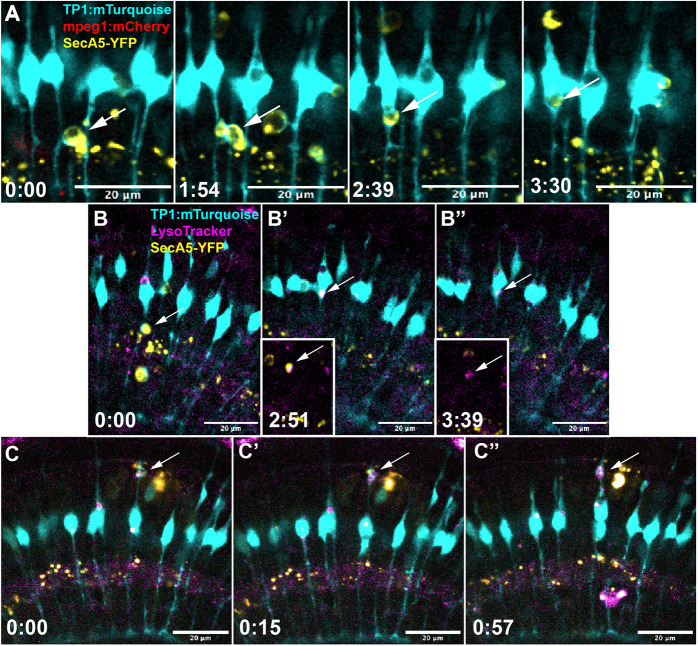
**Müller glia complete engulfment of apoptotic cells.** (A) Time-lapse image stills show the formation of an mTurquoise^+^ phagocytic cup (cyan) around a YFP^+^ dying cell (white arrow) followed by a progression of internalization into the Müller cell body. See Movie 7. (B-B″) Time-lapse stills show the process of Müller glial (cyan) engulfment of a YFP^+^ cell (white arrow). The YFP^+^ cell is brought into the Müller cell body, upon which the compartment fluoresces with the lysosome signal (magenta, co-label in white). Insets show only YFP and magenta coloring to reveal co-localization of the YFP^+^ cell with lysotracker (B′, inset, arrow) then loss of YFP^+^ signal (B″, inset, arrow). See Movie 8. (C-C⁗) In some cases, YFP^+^ cells are engulfed and degraded by Müller glia (cyan) in a region other than the primary cell body. Here, such degradation is shown to occur in the apical process of the Müller cell. Again, the YFP^+^ signal co-labels with the lysosomal reporter (magenta, indicated by white arrow). Time stamps for all panels, bottom left (h:min), are relative to the first frame in the time series.

The transfer of dying cell cargo from MG to microglia, with the cargo terminally engulfed by microglia, is the predominant outcome in dying cell clearance (Fig. 2D). Although less frequently observed, we did note a subset of dying cells terminally engulfed and degraded by MG (Fig. 2F, Fig. 4; Movie 7). In these cases, we observed a complete process of Müller cell extension towards, and around, apoptotic cell bodies coupled with an inward retraction of the process that resulted in an internalization of the dying cell in a putative compartment, with compaction and fading of YFP signal inside of the MG cell body (Movie 7; Fig. 4A).

To further confirm degradation of YFP^+^ targets within MG via lysosomes, we incorporated LysoTracker dye into our live imaging protocol to identify the presence of acidic lysosomal compartments within the cell (Movie 8; Fig. 4B,C). We found that LysoTracker co-localized with vacuolar compartments in MG containing the engulfed YFP^+^ target cell signal, which is then degraded (Movie 8; Fig. 4B-C″). Collectively, we conclude that at least some MG complete terminal engulfment degradation of engulfed dying cells, resolving controversy about such capacity, and indicating that MG are indeed involved in final dying cell clearance in the developing retina.

### Terminal dying cell clearance in microglia versus MG

**Fig. 5.**
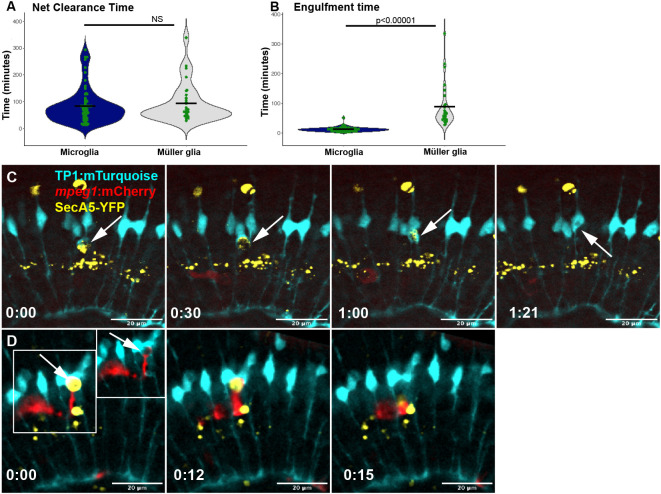
**Quantification of clearance time and engulfment speed by cell type.** (A) Quantification of net clearance time, defined as the time from onset of apoptosis signal to terminal clearance by either microglia or Müller glia (MG). (B) Quantification of engulfment time, defined as the time from the attachment of the glial cell phagocytic cup to the YFP^+^ target cell by either microglia or MG, to terminal engulfment into the glial cell. Results for statistical analysis are shown (NS=not statistically significant in A and *P*-value shown in B); Mann–Whitney *U*-test. Each data point in the violin plots represents the net clearance time (A) or engulfment time (B) for an individual YFP^+^ cell, taken from all recordings. Horizontal line indicates mean and shading indicates distribution of the data points. (C,D) Selected examples of terminal engulfment by MG (C) or microglia (D). In C, time-lapse stills of a Müller glial cell (cyan) completing terminal engulfment of a YFP^+^ cell, with time 0:00 coinciding with the formation of a phagocytic cup around the YFP^+^ cell (arrow). In this example, the Muller glial cell completes engulfment in 81 min. In D, time-lapse stills of a microglial cell (red) completing terminal engulfment of a YFP^+^ cell, with 0:00 coinciding with the formation of a phagocytic cup around the YFP^+^ cell (arrow). In this example, the cell is originally engaged by a Müller cell (cyan, time 0:00, arrow) then retrieved by the microglial cell (red), which completes engulfment in 15 min. Note that this is the same series described for cargo transfer in Fig. 2B. Time stamps, bottom left (h:min), are relative to the first frame in the time series.

Given that we observed both microglia and MG perform terminal engulfment and degradation of YFP^+^ cells, we compared the process by which these cell types complete clearance. We noted the time at which the YFP^+^ cell death reporter was first detected, defining this as the onset of the cell death signal. We then observed the YFP^+^ cell until it was terminally cleared by either a microglial or Müller glial cell, and we defined this as the point in which the YFP signal was displaced from its native site and fully internalized to the phagocyte. The time between the onset of the YFP signal to terminal internalization by the phagocytic cell is defined as the net clearance time. Apoptotic cells had a wide range of net clearance times whether they were cleared by MG or microglia (Fig. 5A), and this did not change significantly based on the cell type performing the terminal engulfment. However, net clearance time does not measure how fast the terminal phagocyte actually clears the cell once it has contacted it. Thus, we defined another subpoint within the time window of the net clearance time, which we defined as the formation and attachment of a phagocytic cup. From the contact by a cellular process, we measured the time elapsed before the YFP^+^ cell body had been displaced from its site and internalized to its respective terminal phagocyte, defining this timeframe as the engulfment time. Of note, we measured time from the formation of the phagocytic cup by the glial cell that completed the terminal engulfment. When we quantified this process for each glial cell type (Fig. 5B), we found a substantial difference between the speed of engulfment by microglia versus MG: microglia averaged ∼12 min to complete terminal engulfment, whereas MG averaged ∼88 min (Fig. 5B). Selected examples of engulfment are shown in Fig. 5C,D.

### Other dynamic and novel Müller cell behaviors

**Fig. 6.**
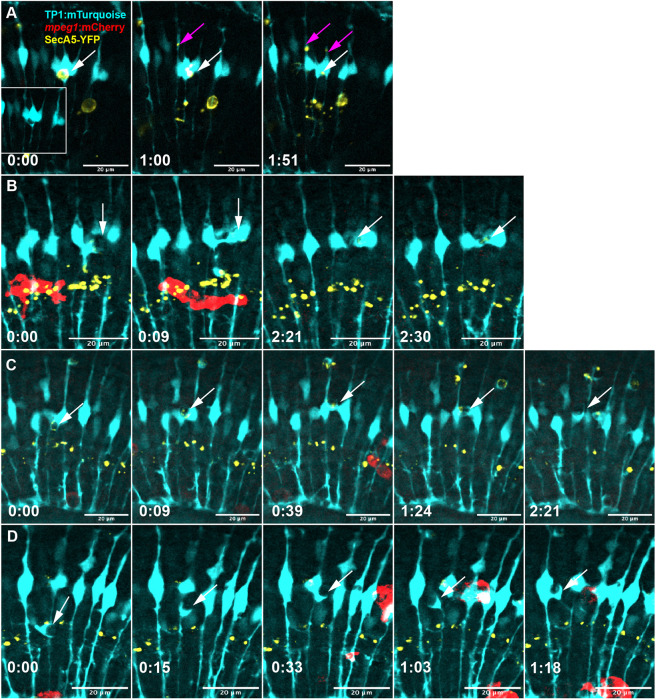
**Other intriguing Müller glia behaviors revealed in real-time.** (A) Müller cell with a YFP^+^ cell (white arrow) fragments the cell and distributes the fragments to adjacent Müller cells (magenta arrows). Inset in A (cyan only) shows the original single compartment around the YFP^+^ cell. See Movies 9 and 10. (B) Müller cell extends a tentacle process around an emerging YFP^+^ apoptotic cell (arrow) and engages with another Müller cell, with both cells engaging with the YFP^+^ target. See Movie 11. (C) An apoptotic cell (YFP^+^, arrow) is engaged by two Müller glia simultaneously. Over time, the cell is captured by one of the initial two cells, but before that is transferred to another cell after another bout of wrestling; see Movie 12. (D) A long extension protruding from a Müller cell (arrow) winds to and from adjacent cells back towards the original cell body over time. See Movie 13. Time stamps in all panels, bottom left (h:min), are relative to the first frame in the time series.

Our time-lapse recordings further revealed several dynamic and intriguing behaviors by MG that, to our knowledge, have not previously been described. One such noted observation is the fragmentation or splitting of SecA5-YFP^+^ cargo coupled with distribution to other MG (Movies 9 and 10; Fig. 6A). In this case, a single YFP^+^ target cell was moved to a single Müller cell body before it was fragmented and distributed among multiple adjacent Müller cells (Movies 9 and 10). In addition, we observed examples in which at least two MG reach for the same YFP^+^ target, resulting in an apparent ‘wrestling’ or ‘tug-o’-war’ between these two cells for the cargo (Fig. 6B; Movie 11). Similarly, this wrestling may be coupled with lateral passing of cargo between multiple Müller cells (Movie 12; Fig. 6C). In addition to behaviors of engulfment and cargo management, we observed the extension of remarkably long, dynamic and winding cellular processes from the body of Müller cells (Movie 13; Fig. 6D).

### Clearance of phosphatidyl serine-positive puncta in the inner retina

**Fig. 7.**
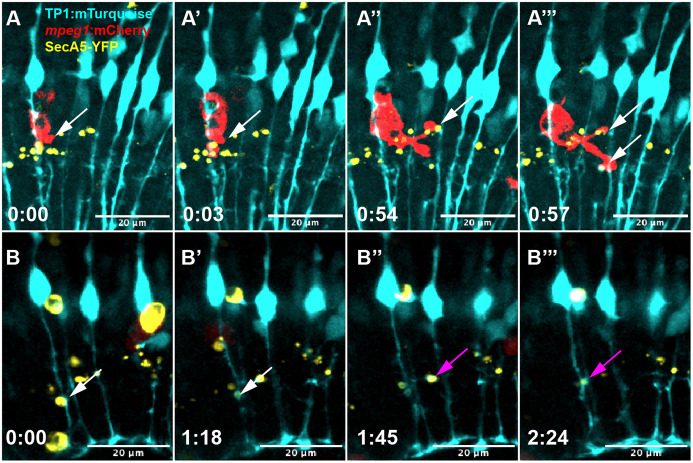
**Microglia and Müller glia engulf PtdSer^+^ puncta in the inner plexiform region.** (A-A‴) A microglial cell (red) engulfs distinct YFP^+^ puncta with multiple processes extended from the cell body (white arrows). (B-B‴) Müller glia (cyan) also engulf YFP^+^ puncta using processes (arrow). The white arrow shows one YFP^+^ puncta engulfed, then the same Müller cell engulfs a second YFP^+^ puncta (magenta arrow). Time stamps in all panels, bottom left (h:min), are relative to the first frame in the time series. See Movie 14. Note that Movie 13 (described in Fig. 6D) and Movie 14 partially overlap temporally.

As described in our previous study (Blume et al., 2020), we observed numerous puncta (less than 3 μm diameter) from the SecA5-YFP reporter visualized in a location consistent with the inner plexiform layer of the developing retina (basal to the MG cell body yet apical to the MG end-feet; Fig. 7). The presence of these SecA5-YFP^+^ puncta indicate that PtdSer^+^ regions exist in the inner retina. These YFP^+^ puncta became visible independent of larger YFP^+^ cell bodies, and we observed that both microglia and MG were active in the engulfment and clearance of the PtdSer^+^ puncta (Movie 14; Fig. 7A-B‴). MG engulf these SecA5-YFP^+^ puncta with fine process extensions and appear to internalize these puncta more efficiently compared with larger YFP^+^ cell bodies (Movie 14, right; Fig. 7A,B).

### Expression of selected phosphatidyl serine-recognizing receptors by microglia and MG

Exposed PtdSer on the surface of apoptotic cells serves as a ligand for PtdSer-binding phagocytic receptors on phagocytes. A wide array of phagocytic receptors exists and have, to varying extents, been characterized using various models or conditions of phagocytosis (Naeini et al., 2020). The existing body of work concerning microglial phagocytic receptors has largely been concentrated in mammalian models and focused on the microglia of the brain (VanRyzin, 2021). Also in zebrafish, such work has focused on the brain (Mazaheri et al., 2014). In contrast, expression of phagocytic receptors by MG is less well known, though recognition of PtdSer is likely involved (Nomura-Komoike et al., 2020; Thiel et al., 2022b). In addition, phagocytic receptors expressed by zebrafish retinal microglia have not been directly examined. We therefore examined expression of phagocytic receptors by microglia and MG during zebrafish retinal development, to suggest those which could be important for phagocytic behaviors observed in our recordings.

We used hybridization chain reaction fluorescent *in situ* hybridization (HCR-FISH; Choi et al., 2018) to probe expression of mRNA encoding selected phagocytic receptors known to bind to PtdSer either directly or through bridging molecules (Naeini et al., 2020). These receptors were selected based on expression in RNA-seq datasets probing zebrafish microglia and MG (Mitchell et al., 2019; Oosterhof et al., 2017; Sifuentes et al., 2016) and experimental reports (Anderson et al., 2022; Hsiao et al., 2019; Mazaheri et al., 2014; Nomura-Komoike et al., 2020). The selected transcripts encode *axl*, *mertka*, *havcr1*, *timd4*, *itgam.1*, *itgb2* and *lrp1aa*. The products of the genes *mertka* and *axl* belong to a family of receptor tyrosine kinase proteins known as the TAM family (Lemke, 2017), which bind to PtdSer via Gas6 (Lemke, 2017). Zebrafish *havcr1* and *timd4* encode proteins within the T-cell immunoglobulin domain-containing (TIM) family and bind PtdSer directly (Naeini et al., 2020; Xu et al., 2016); these genes have orthology to mammalian *TIM4* (*TIMD4*) and *TIM1* (*HAVCR1*) (Xu et al., 2016). *Itgam.1* and *itgb2* are predicted orthologs of human/mouse *ITGAM* and *ITGB2*, which together in mammals form the complement receptor 3 (CR3) (Bader et al., 2021). Binding of CR3 is bridged with PtdSer via C1q (Naeini et al., 2020). LRP1 can act as a phagocytic receptor (Donnelly et al., 2006) and was upregulated by MG responding to retinal damage (Sifuentes et al., 2016).

**Fig. 8.**
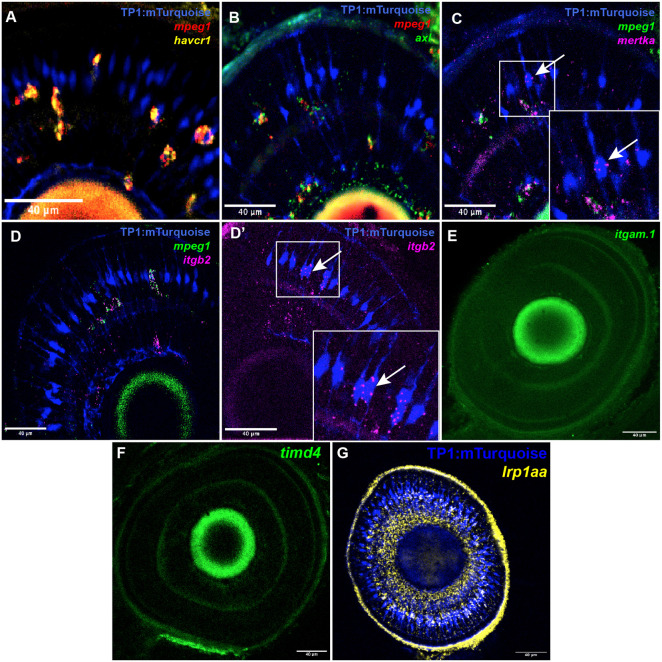
**Expression of selected phagocytic receptors in wild-type zebrafish retinas during development.** (A-G) HCR-FISH was used to visualize transcripts for the indicated receptors in retinas expressing markers for Müller glia (MG; *TP1:mTurquoise*, blue) and microglia (*mpeg1*:mCherry/*mpeg1* transcript) at 3 dpf. Images show selected *z*-planes acquired from whole embryonic eyes/retinas. (A) Expression of *havcr1* was limited to microglia and strongly expressed. (B) The receptor *axl* was also strongly expressed in microglia cells. (C) Expression of *mertka* was found in both microglia (green and magenta co-localization) and MG (arrow), though microglia displayed stronger signal, and expression in MG appeared to be heterogeneous, with only some MG with detectable transcript label in the cell body. (D,D′) Complement receptor component *itgb2* was strongly expressed in microglia (D) in addition to some expression by MG (D′, arrow), albeit heterogeneously and in varying amounts. (E,F) Transcripts for *itgam.1* (E) and *timd4* (F) were not appreciably detected. (G) Transcripts for *lrp1aa* did not appear to be restricted to glial cells. Scale bars: 40 µm.

We visualized mRNA *in situ* in the developing zebrafish retina at 3 dpf, coinciding with the endpoint of our time-lapse recordings, in combination with microglia- (*mpeg1*:mCherry and/or *mpeg1* transcript) and MG- (*TP1*:mTurquoise) specific markers. We found that microglia expressed multiple PtdSer-recognizing receptors, with strong expression of *axl*, *havcr1*, *mertka* and *itgb2* (Fig. 8A-D)*.* The expression of *itgam.1* was not convincingly detected (Fig. 8E). It is possible that, in zebrafish, the complement system does not directly mirror that seen in mammals and may employ a different integrin-α binding partner for *itgb2*, or *itgb2* is used differently. In contrast to the results seen for microglia, we found that MG expressed only two of these selected PtdSer receptors (*mertka* and *itgb2*; Fig. 8C,D′); this expression was apparently heterogenous, and the expression did not match the level found in microglia. We did not detect appreciable expression of *timd4* in any cells in the retina at this timepoint (Fig. 8F), whereas expression of *lrp1aa* appeared to be nearly ubiquitous in the retina (Fig. 8G) and was not restricted to any cell type, glia or other. Collectively, these results indicate redundant and strong expression of multiple PtdSer receptors by microglia, with more limited expression by MG. Such expression patterns are consistent with the dominant role of microglia in terminal engulfment and could underlie the basis for cargo transfer from MG to microglia or to other MG more competent for engulfment.

## DISCUSSION

Before this work, simultaneous visualization of MG, microglia and dying cells in real-time in the intact developing retina had not been described. Though we have examined microglial clearance of dying cells (Blume et al., 2020) and others have recorded microglial clearance of ablated rod photoreceptors (White et al., 2017) in developing retinas, real-time imaging of the MG is limited to their developmental differentiation (MacDonald et al., 2015). Our current study further exploits the advantages of the zebrafish for live time-lapse imaging to observe behaviors of MG and microglia together in apoptotic cell clearance during retinal development. Our recordings reveal a complex and previously unreported set of interactions between MG and microglia and between neighboring MG. The results of our study reinforce the paradigm of microglial dominance in phagocytosis; however, we additionally reveal that MG are surprisingly dynamic and active in responding to dying cells and observed that both glial cell types interact with most apoptotic cells before their clearance. Not only do the MG most frequently make the first contact with the apoptotic cell, but they also often form an initial phagocytic cup-like structure around the target, after which the target is then often engulfed and terminally degraded by microglia following a transfer process. Despite the frequent ‘switch’ of phagocyte for terminal engulfment after initial contact, MG indeed fully execute phagocytosis for a subset of dying cells. Collectively, these real-time observations resolve seemingly conflicting reports from fixed tissue by revealing transient yet common interactions of both glial cell types with a target cell and resolving that both cell types do indeed complete phagocytic engulfment.

In between the beginning and end of the apoptosis-phagocytosis sequence, an average of over 60% of all phagocytic events observed in our recordings showed a ‘switch’ of phagocyte from MG to microglia. At times, microglia were engaging and commandeering the apoptotic cell before the MG could completely enwrap the cell yet, more commonly, there was a formed cup-like structure around the dying cell at the time of microglial engulfment. It is not known whether this is a coordinated or unilateral process, nor whether the initial Müller response and contact may act as a support mechanism for a stressed cell before terminal phagocytosis rather than an attempt at phagocytosis itself. We did not observe examples of transient PtdSer exposure/YFP reporter and restricted our analysis to YFP^+^ cells that were engulfed during the recordings. Thus, we cannot exclude transient contact of MG with stressed cells that we could not visualize. Furthermore, the detection of Müller glial cell reporters, both cytoplasmic and membrane tagged, being taken from MG to microglia with the apoptotic cell raises new questions over the structure of the compartments formed and potential biological basis for such transfer, given that we did not observe apoptotic MG in our recordings. In particular, such observations suggest that the entire Müller cell cup structure may be engulfed by microglia in addition to the apoptotic cell target. Whether material transfer occurs and is a biologically important process remains to be determined. However, it is worth noting that intercellular transfer from photoreceptors to MG (Hutto et al., 2023), and from macrophages to other cells (Kidwell et al., 2023; Roh-Johnson et al., 2017), have been documented. Nonetheless, the observations from our recordings support that both a ‘strong-armed’ approach by microglia, in which microglia outcompete MG for the apoptotic cell to pull it away, may be possible in addition to a coordinated process in which one or both cells execute a regulated signaling process for the exchange of the YFP^+^ cells, both of which are yet to be investigated.

Our findings indicate that sensing of dying cells and their phagocytosis, when executed, is an active process on the part of MG rather than passive collapse around already enwrapped neurons. MG actively extended cellular processes towards PtdSer-exposing cells, often times before detection of the cell death reporter on the targets. The molecular sensing of apoptotic targets by MG remains unknown but may follow suit with regards to apoptosis-sensing mechanisms found in macrophage cells including, but not limited to, purinergic sensing (Blume et al., 2020), fractalkine (Sokolowski et al., 2014) and lysophosphatidylcholine (Lauber et al., 2003). After contact, phagocytic cups frequently formed, and in scenarios when the Müller cell completed engulfment, this was followed by a subsequent retraction inward. Our usage of a lysosome tracker in real-time confirmed the active degradation of target cells by MG upon lysosomal fusion. This is consistent with our previous work showing Rab5^+^ endocytic vesicles and increased lysosomal staining inside of MG in fixed tissue samples from microglia-deficient retinas, where MG show compensatory phagocytic activity (Thiel et al., 2022b). In the mouse brain, several reports indicate the capacity of astroglia to perform phagocytosis of apoptotic cells and their debris (Damisah et al., 2020; Konishi et al., 2020, 2022; Morizawa et al., 2017). Given that studies in the invertebrate *Caenorhabditis elegans*, which lack professional phagocytes like macrophages, served as a foundation for our knowledge of genes regulating the molecular process of dying cell clearance (Conradt et al., 2016; Lukácsi et al., 2021), it is not entirely surprising that vertebrate neuroglia display such capacity. It is suggested that vertebrates developed specialization for managing phagocytic load by professional phagocytes such as macrophages/microglia possibly to reduce load on neuroglia, to in turn allow more specialized and complex neuroglial functions in the vertebrate central nervous system. Such an idea is consistent with signs of reactivity from MG due to increased phagocytic load when microglia are absent (Thiel et al., 2022b).

In the case of microglial phagocytosis of apoptotic cells, we observed a single microglial cell manage an entire cellular target in terms of acquisition and degradation. On the other hand, MG exhibited apparent varied behaviors, strategies or mechanisms for handling phagocytic cargo. In some cases, engulfment was not completed by a sole MG, but rather by a consortium that involved more than one Müller cell either wrestling for the cell or partitioning the cell in a way in which more than one cell received a portion to ingest. In some cases, Müller cells apparently passed the targets to neighboring Müller cells. Yet, in other cases, a single Müller cell would complete the engulfment of the cell. In this light, it could be said that microglial phagocytosis is more uniform in its procession than Müller glial phagocytosis. In contrast, for PtdSer^+^ puncta, MG appeared to efficiently engulf these smaller targets. This may suggest that heterogeneity in phagocytic capacity exists for Müller cells, and this variable capacity for engulfment could exist at a cellular level and depend on the size of the target.

In addition to the relative phagocytic loads, there were other factors that separated the phagocytic characteristics of microglia and MG. One such factor is the relative time of engulfment. Microglia were noticeably faster in completing the clearance of apoptotic cell bodies upon the attachment of a phagocytic cup. This process took significantly longer for MG. This difference may be due to the greater ability of microglia to adapt their morphology for process extension and migration via rapid cytoskeletal rearrangements, whereas MG are more or less conformed to a fixed radial shape, with more steps required for process extension and phagocytic cup formation. We did observe many times that MG are capable of extending dynamic processes from various parts of their cell body. An additional difference could derive from the expression of PtdSer-recognizing receptors. Of the selected receptors investigated, microglia strongly express at least four of them (*axl*, *mertka*, *itgb2*, *havcr1*), while MG were limited to the expression of *merkta* and *itgb2.* Yet even for those two genes, expression strength did not visibly match that of microglial cells and expression amongst MG was not apparently uniform. Heterogeneity in *mertka* and *itgb2* expression by MG is also represented in publicly available single cell RNA-seq datasets (Hoang et al., 2020) and could explain apparent differences in phagocytic capacity between Müller cells. Further, the difference in expression of PtdSer receptors between the two glial cell types may play a role in determining the nature of the phagocytic dominance of microglia, and these receptors may play a potential role in cargo switching between the two cell types in which microglia remove the target from the Müller glial phagocytic cup.

Another interesting observation, though not the intended focus, was the clearance of PtdSer^+^ puncta in an area that is consistent with the inner plexiform layer, a laminated region of synaptic connections in the inner retina. In our recordings, PtdSer^+^ puncta were engulfed by both microglia and MG. Although our studies do not explicitly identify the nature of this puncta, and this signal may simply represent debris remaining after neuronal bodies are cleared, existing literature suggests that localized PtdSer exists on synapses and could be recognized by microglia for synaptic refinement (Györffy et al., 2018; Kurematsu et al., 2022; Park et al., 2021; Rueda-Carrasco et al., 2023; Scott-Hewitt et al., 2020).

Collectively, our recordings reveal tremendous insight into previously unknown behaviors of the MG, some of which suggest functional roles that have not yet been explored. Such dynamic and interactive behaviors should change our view of how the MG function in the vertebrate retina, and better inform our understanding of these glial cells in health and disease.

## MATERIALS AND METHODS

### Animals

All procedures were in compliance with protocols approved by the Institutional Animal Care and Use Committee at the University of Idaho. Adult zebrafish were housed in an aquatic environment at 28.5°C with monitored, recirculating system water and an automated diurnal light cycle of 14 h light and 10 h dark, according to Westerfield, (2007).

Zebrafish embryos were collected from breeder pairs with spawning time corresponding to the onset of light in the housing environment. Embryos were collected and housed in an incubator at 28°C with a final concentration of 0.06% N-Phenylthiourea (PTU) added to the water to prevent the development of melanin pigment. Water and PTU solutions were refreshed daily. At an age of at least 2 dpf, embryos were sorted for positive expression of each transgene; embryos expressing all desired transgenic reporters were selected for use in the experiments. Before use in experiments, the chorion was manually removed with fine tip forceps as necessary. Zebrafish cannot be sexed until reproductive age, therefore sex of embryos is unknown.

### Transgenic lines

The *TP1:mTurquoise* (uoi2523Tg) transgenic line used to visualize MG was generated in-house using a Tol2 transgenesis construct kindly gifted by Ryan MacDonald (University College London, UK) based on MacDonald et al. (2015). The *TP1:mCherry-CAAX* transgenic line was described in our previous work (Thiel et al., 2022b). Microglia were visualized using the pre-existing transgenic lines *mpeg1*:mCherry (gl23) or *mpeg1*:GFP (gl22) (Ellett et al., 2011), originally obtained from the Zebrafish International Resource Center and maintained at the University of Idaho. Apoptotic cells were visualized using the TBP:Gal4;UAS:SecA5-YFP transgenic line (referred to throughout this paper as SecA5-YFP) (van Ham et al., 2010) originally gifted by Randall Peterson (University of Utah, UT, USA) and maintained at the University of Idaho. We generated the transgenic line *bact2*:SecA5-mTagBFP (uoi2518Tg) using Gateway cloning to recombine p5E-*bact2* (obtained from the 2007 Tol2 kit) (Kwan et al., 2007), pME-secAnxaV-NS (Morsch et al., 2015; Addgene plasmid #67718), and p3E-mTagBFP (Don et al., 2017; Addgene plasmid #75175) into pDEST-Tol2CG2 (Tol2 kit). The size and orientation of the final construct was validated by restriction enzyme digestions. For in-house generated transgenic lines, single blastomere-stage zebrafish embryos were microinjected with ∼0.5-1 nl of DNA construct mixed with Tol2 mRNA (25 ng/µl final concentration). F0 founders were selected based on expression of the transgene as embryos, grown to adults, then out-crossed with non-transgenic wild-type fish to F1 showing germline inheritance of the transgene. F1 were then again out-crossed to obtain stable lines at F2 or later generations, with ∼50% transgene segregation upon out-cross to non-transgenic partners. Zebrafish carrying multiple transgenic reporters were obtained by crossing stable transgenic lines over two to three generations. Zebrafish lines used in this study can be obtained from the Zebrafish International Resource Center or via request and plasmids can be obtained from Addgene or via request.

### Live-time lapse imaging

For time-lapse recordings, embryos were anesthetized in a solution of water, 0.06% PTU and 0.016% Tricaine (MS-222). Once anesthetized, embryos were suspended in liquified, low-melting temperature agarose (1% agarose prepared in fish system water) and overlayed onto a coverslip-bottom dish (No. 1.0 thickness, 35 mm, MatTek Corporation). Embryos were oriented so that they were on their side with one eye against the coverslip. The agarose gel pad, which then solidified, was overlayed with water/PTU/MS-222 solution to ensure continued anesthesia and pigment inhibition of the fish during imaging.

Embryos on the coverslip dishes were transferred to a climate-controlled chamber (Okolab) maintained at 28°C on the microscope stage. Images were acquired using a Nikon Andor spinning disk confocal microscope connected with a BSI Express 16-bit sCMOS camera. Imaging was performed using a CFI APO LWD 40× water immersion 1.15 NA λ S DIC N2 objective. An immersion compound (Zeiss Immersol™ W) with a refractory index of 1.334 was placed on the objective in lieu of water to avoid evaporation over the imaging session. The region for imaging was selected based upon the visibility of entire radial MG, with an average field size of ∼180 µm×200 µm. Imaging depth spanned ∼40 µm, with 3 µm *z*-steps. Each imaging session lasted 10 h with 3-min time intervals between each capture, spanning a total of 12-15 *z*-stacks. Embryos were selected for imaging and analysis by display of strong heartbeat and circulation throughout the tissue both before and after the imaging session. The mTurquoise, YFP and mCherry fluorophores were excited with 445 nm, 514 nm and 561 nm lasers, respectively, with a Chroma 89006 (eCFP, eYFP, mCherry) dichroic mirror. For imaging GFP, mCherry and BFP, excitation used 488 nm, 546 nm and 405 nm, respectively, and the Chroma 89000 Sedat Quad dichroic mirror.

### Image viewing and processing

Time-lapse image stacks were viewed with both Nikon Elements software and FIJI. Images selected for figures were derived from selected time points in a recording and converted from their original 16-bit format into a RGB tiff format after image adjustments using FIJI. 3D renderings for selected movies were performed using Nikon Elements through projection in volume view. Movies were made by saving select time series as ‘.avi’ files with jpeg compression. Images were assembled into figure arrangement and annotated in Adobe Illustrator. Movies were annotated using FIJI.

### Quantification of phagocytic events in time-lapse recordings

Images were quantified manually using ImageJ software. Counts were made by noting phagocytic events during the recording by examining individual *z*-stacks and maximum *z*-projection images to avoid overcounting. Phagocytic events for apoptotic cells were defined as the terminal engulfment of SecA5-YFP^+^ bodies (of size 4-7 μm in diameter) by a glial cell, and only events that fit such criteria were analyzed. A report of the number of YFP^+^ cells tracked and counted in each recording is provided in Table S1. Each event was studied in terms of the glial cell that contacted the cell first (by watching the image series before the terminal engulfment) and the glial cell that completed terminal engulfment of the YFP^+^ cell. Each cell that was engulfed during a recording session was then scored for first cell type to contact it (MG or microglia), visual evidence of transfer from one cell type to the other, and the final cell type to terminally engulf it (MG or microglia). The transfer of cargo was defined as YFP^+^ cells that were initially engaged by MG but were subsequently engulfed by microglia.

The net clearance time was recorded as the elapsed time beginning at the timepoint at which a YFP^+^ cell appeared in the recording until the timepoint at which it was terminally cleared by a glial cell. This elapsed time and the phagocyte cell type (MG or microglia) was recorded for each YFP^+^ cell analyzed. YFP^+^ cells already present at the beginning of the recording, or which were not terminally cleared by the end of the recording, were not analyzed because true elapsed clearance time could not be determined. The engulfment time for each glial cell (MG or microglia) was determined as the elapsed time beginning at the time the glial cell formed a phagocytic cup around the YFP^+^ cell to the timepoint at which it successfully removed the apoptotic cell from its native site and internalized it. The engulfment time elapsed and the terminal phagocyte cell type (MG or microglia) was recorded for each YFP^+^ cell analyzed. Cells already being internalized by phagocytes before the beginning of the recording, or those not completed by the end of the recording, were not counted in the analysis. Graphical presentation of quantifications was performed using RStudio.

### Lysotracker™ immersion

To visualize lysosomes in live zebrafish retinas, we prepared embryos the same as described above with an additional step before mounting. In this step, a 1 µM solution of LysoTracker™ Deep Red (Life Technologies, L12492; made from 1 mM stock in zebrafish system water) was prepared with 0.06% PTU. Embryos were placed in this solution and incubated in the dark at 28°C for 30 min. Following incubation, embryos were washed twice with system water to remove excess LysoTracker™ from the surface of the embryos. Embryos were then placed in a new solution of system water and 0.06% PTU before suspension in the agarose pad. Embryos were oriented in the agarose pad and mounted on the coverslip dish as described above. The agarose pad containing the embryos was overlayed with 1 µM LysoTracker™ plus 0.06% PTU in fish system water. For imaging of LysoTracker™ Deep Red, a 640 nm (far-red) excitation laser was used.

### Fluorescence *in-situ* hybridization

RNA fluorescent *in-situ* hybridization was performed using hybridization chain reaction (HCR) protocol, reagents and probes from Molecular Instruments (Choi et al., 2018) and as performed previously to examine microglia-expressed transcripts in zebrafish embryos (Thiel et al., 2022a). Probes were custom designed by Molecular Instruments based upon the NCBI accession number (Table S2) of each transcript. At 3 dpf, embryos were fixed in a freshly made solution of 4% paraformaldehyde (PFA) in 1× phosphate buffered saline (PBS). Before fixation, the PFA/PBS solution was cooled to 4°C to prevent autofluorescence in the samples. Once in the fixative solution, the embryos were incubated for 24 h at 4°C with constant rocking. After fixation, the PFA solution was removed, and the embryos were washed three times with RNAse-free 1× PBS solution for 5 min per wash. Samples with transgenic fluorescent reporters (e.g. *TP1:mTurquoise*) were protected from exposure to light to prevent destruction of the fluorophore. Following washes, embryos were washed four times with 100% methanol for 10 min each, followed by one 50-min wash in 100% methanol. Embryos were stored in a fresh solution of 100% methanol and stored at −20°C at least overnight before use.

To begin the hybridization procedure, embryos were rehydrated in a graded series of methanol: PBS-Tween (1× PBS+0.1% Tween 20; PBST) washes for 5 min each. The order of the graded series (in ratio of methanol:PBST) was 75:25, 50:50, 25:75 and finally 100% PBST. Following the rehydration of the embryos, they were treated with 1 ml of a 10 µg/ml solution of proteinase K for 10 min at 37°C. Proteinase K solution was removed, and embryos were rinsed briefly with PBST twice at room temperature. This was followed by post-fixation with 4% PFA in PBS for 20 min at room temperature. Embryos were then washed five times with 1× PBST for 5 min each. PBST was replaced by probe hybridization buffer that had been pre-heated to 37°C, and embryos were pre-hybridized for 30 min at 37°C. A solution of probes was made using 500 µl of probe hybridization buffer and 2 pmol of each probe, with appropriate combinations for multiplexing. After pre-hybridization, the solution was removed and embryos were incubated with the probes in hybridization solution for 12-16 h at 37°C in a hybridization oven.

Following the hybridization, probe solutions were removed and embryos were washed with probe wash solution four times for 15 min each at 37°C, followed by two washes with 5× saline sodium citrate-0.1% Tween-20 (SSCT) buffer for 5 min each. Amplification was performed by incubation in probe amplification buffer for 30 min at room temperature, followed by an overnight incubation (>12 h) with amplifier solution. Then 30 pmol of each hairpin (see Table S2) was snap cooled by incubation at 95°C for 90 s in a thermocycler, then cooled to room temperature in the dark for 30 min. Snap-cooled amplifiers were added to amplification buffer at room temperature as appropriate for multiplexing. The pre-amplification buffer was replaced with the hairpin mixture and incubated for 12-16 h at room temperature, protected from light. Hairpins were removed and embryos were washed with SSCT five times, with DAPI added at 1:1000 in the final wash, then stored in 1× PBS at 4°C until imaging.

The embryos/eyes were subsequently mounted either with or without dissection of the eyes from the body and suspended in glycerol on a 22×60 mm microscope coverslip, using two smaller 22.5×22.5 mm coverslips to act as spacers, then covered and sealed with another 22×60 mm coverslip. Samples were imaged using either 20× air objective or the 40× water-immersion objective lens (described above) on the Nikon Andor spinning disk confocal microscope described above with 2 µm *z*-stacks. Images were collected using Nikon Elements and subsequently processed using ImageJ.

### Statistical information

For comparison of proportion data from time-lapse recordings (Figs 1E and 2E,F), we first used Fisher's Exact test to compare the proportion of events by cell type across each recording finding in which these proportions were maintained (*P*>0.05). We then used binomial proportion test to compare the proportion of events by cell type (first contact, contact type and terminal engulfment). For pairwise comparison of net clearance and engulfment time (Fig. 5A,B), data was non-normal with unequal variances, and a Mann–Whitney *U*-test was selected. For all statistical analyses, a *P*-value below 0.05 was considered statistically significant, and *P*-values are reported in the figures.

## Supplementary Material

10.1242/develop.202407_sup1 Supplementary information

Table S1. Counts of analyzed events in unique recordings from TP1:mTurquoise;SecA5-YFP;mp eg1:mCherry triple transgenic larvae.
