# Supplementary material for: Intercellular contact and cargo transfer between Müller glia and to microglia precede apoptotic cell clearance in the developing retina
Source: Development. 2024 Jan 4;151(1):dev202407. doi: 10.1242/dev.202407 (PMC10820749; doi:10.1242/dev.202407)
Supplement: Supplementary information [file develop-151-202407-s1.pdf]

Table S1. Counts of analyzed events in unique recordings from TP1:mTurquoise;SecA5-YFP;mpeg1:mCherry triple transgenic larvae.

Available for download at
https://journals.biologists.com/dev/article-lookup/doi/10.1242/dev.202407#supplementary-data

Table S2. NCBI accession number, probe set size, and hairpins used in HCR in situ hybridization.

Transcript	NCBI Accession Number	Probe set size	Amplifier format/fluorophore
mpeg1	NM_212737.1	17	B1/546, 488; B3/546,488
mertka	XM_002664231.6	20	B2/647
axl	XM_017351109.2	20	B3/AF488
havcr1	NM_001002434.1	18	B3 AF546
itgb2	XM_680920.7	20	B2/647
itgam.1	XM_009306407.4, XM_009306408.3, XM_005156007.4, XM_009306409.3 (probes will detect all)	17	B1/488
timd4	NM_001122617.1	19	B3/AF488
lrp1aa	XM_021479679.1	20	B1/AF546

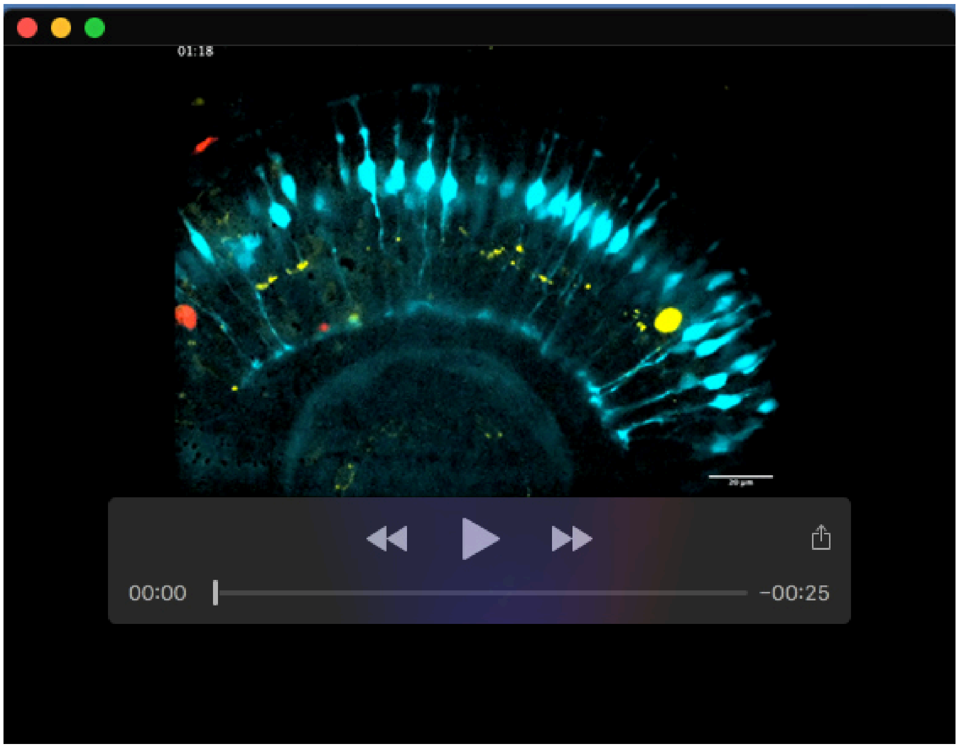

Movie 1. Overview of developmental cell death in the retina. Selected z-stacks (4) projected to represent the dynamics typical of a 10-hour live imaging experiment. Müller cells (turquoise), microglia (red), and apoptotic cells (yellow).

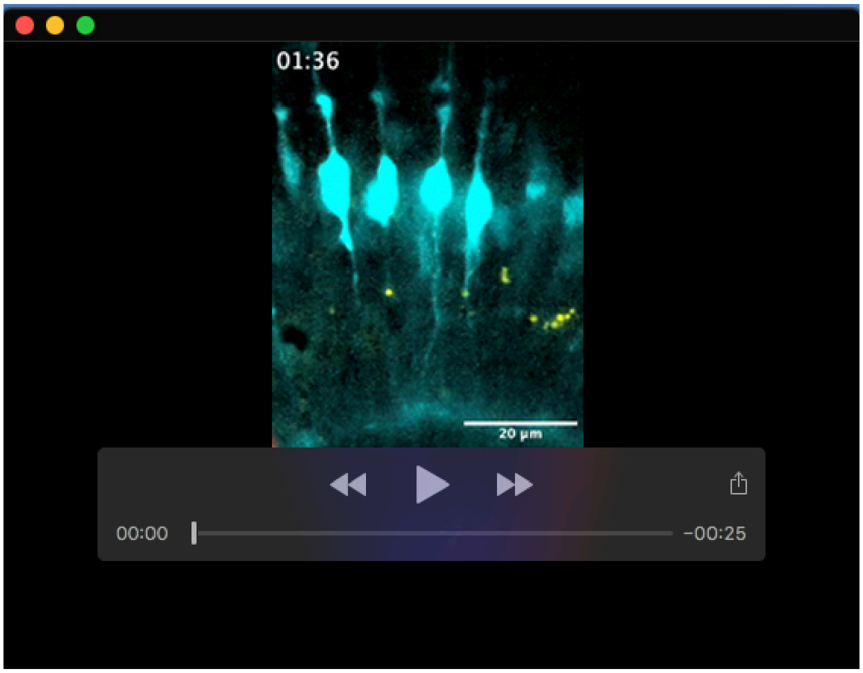

Movie 2. Müller cell initial contact with an apoptotic cell. An apoptotic cell (yellow) emerges and is subsequently contacted by a Müller glia cell (turquoise) which begins moving the cell.

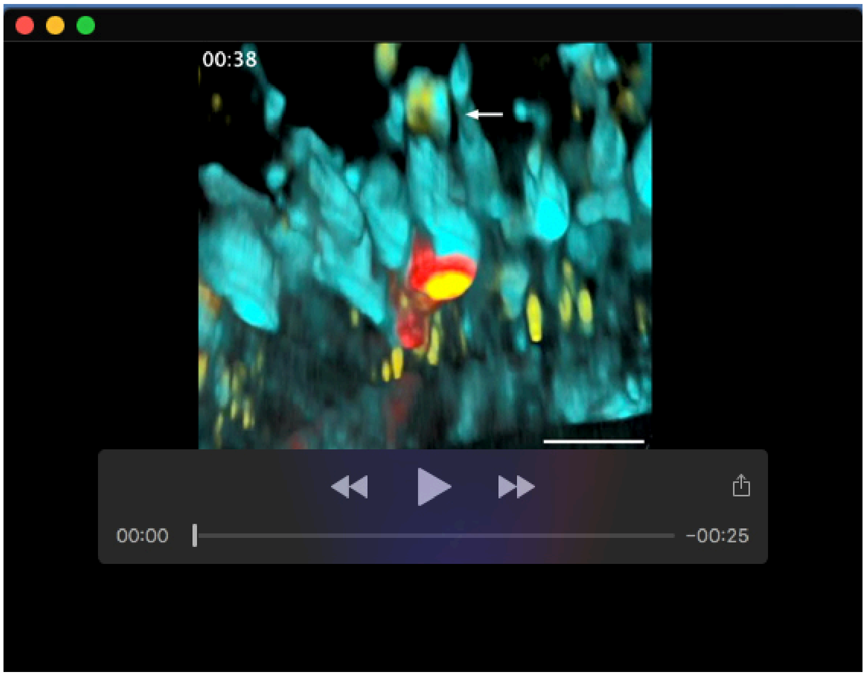

Movie 3. Phosphatidyl-serine+ cargo transfer from Müller glia to microglia-Example I. 3-dimensional projection made from z-stacks shows the appearance of an apoptotic cell (YFP+) in the outer retina (yellow cell, white arrow). The cell is enwrapped by Müller cell processes (turquoise) but is transferred to a microglia cell (red) for terminal engulfment. Scale bar = 20 µm. Pertains to Figure 2A.

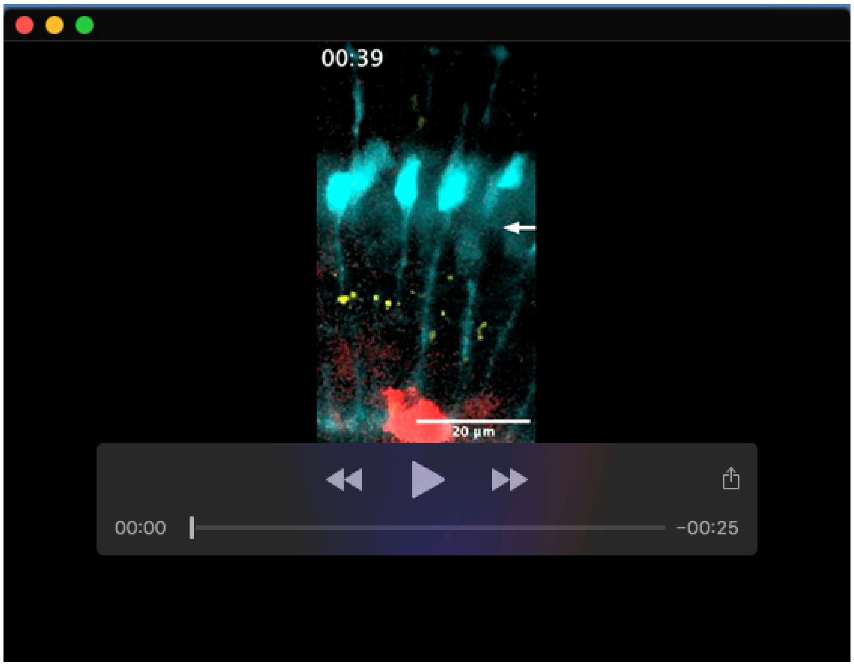

Movie 4. Phosphatidyl-serine+ cargo transfer from Müller glia to microglia-Example II. An apoptotic cell appears (yellow cell, white arrow) and a phagocytic cup-like structure is formed by Müller glia (turquoise) around the cell, but the cell is transferred to and terminally engulfed by microglia (red). Pertains to Figure 2B.

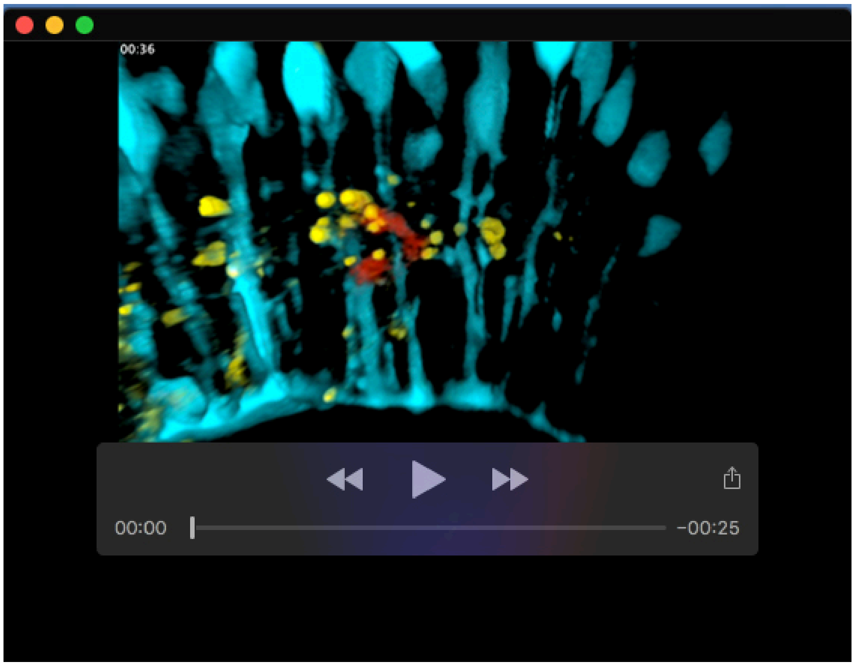

Movie 5. Müller cell cytoplasmic reporter in microglia cell. 3-dimensional projection shows a microglia cell (red) engaging a nascent apoptotic cell signal (yellow). During the interaction, there is Müller cell cytoplasmic reporter (turquoise) visualized in microglia compartments (white arrows) coinciding with the engulfment activity. Scale bar = 20 µm. Pertains to Figure 3A.

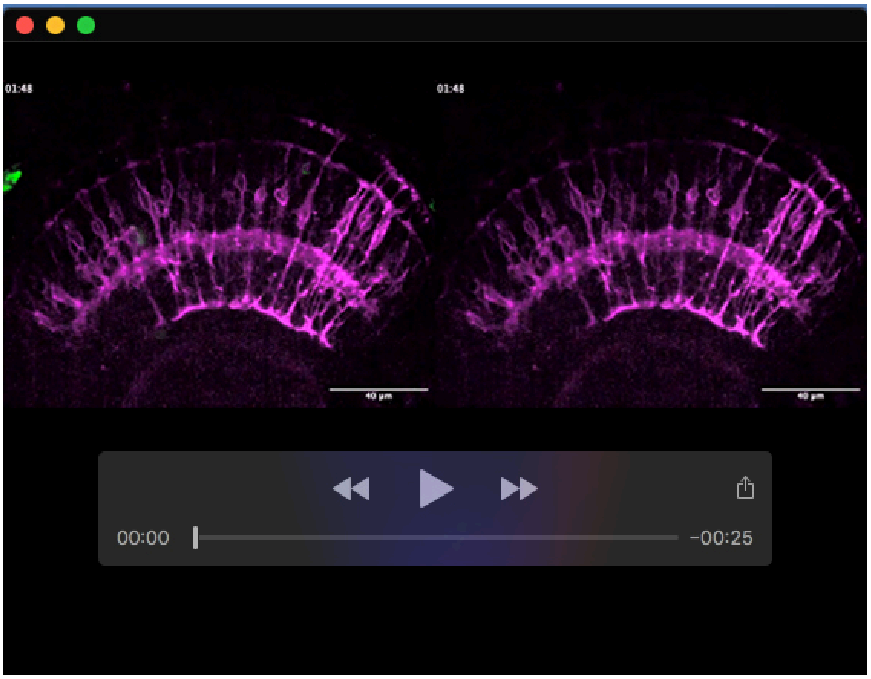

Movie 6. Müller cell membrane reporter in microglia cells. (Left) Projection of selected z-stacks show Müller cell membrane reporter (magenta) localizing with vacuolar compartments within microglia cells (green). (Right) The same movie showing a single channel to visualize the Müller cell membrane reporter moving throughout the retina coincident with microglia. Arrows point to select instances of Müller cell membrane reporter-microglial vacuolar co-localization. Pertains to Figure 3B.

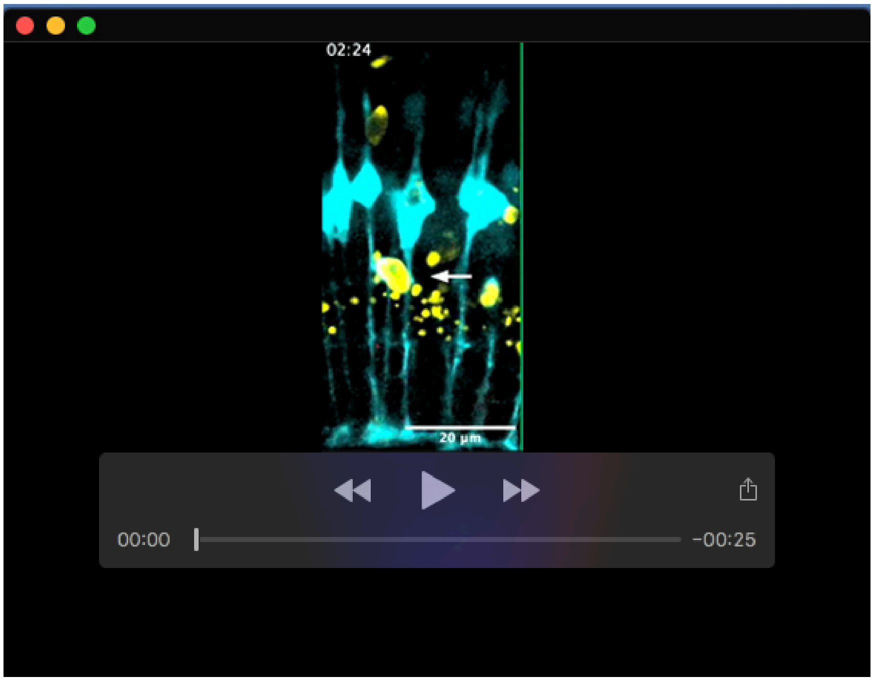

Movie 7. Müller cell performing phagocytosis of an apoptotic cell. A phagocytic event is shown in which a Müller glia cell (turquoise) engages and completes the engulfment of an apoptotic cell (yellow cell; white arrow). The cell is trafficked into the cell body of the Müller cell. Pertains to Figure 4A.

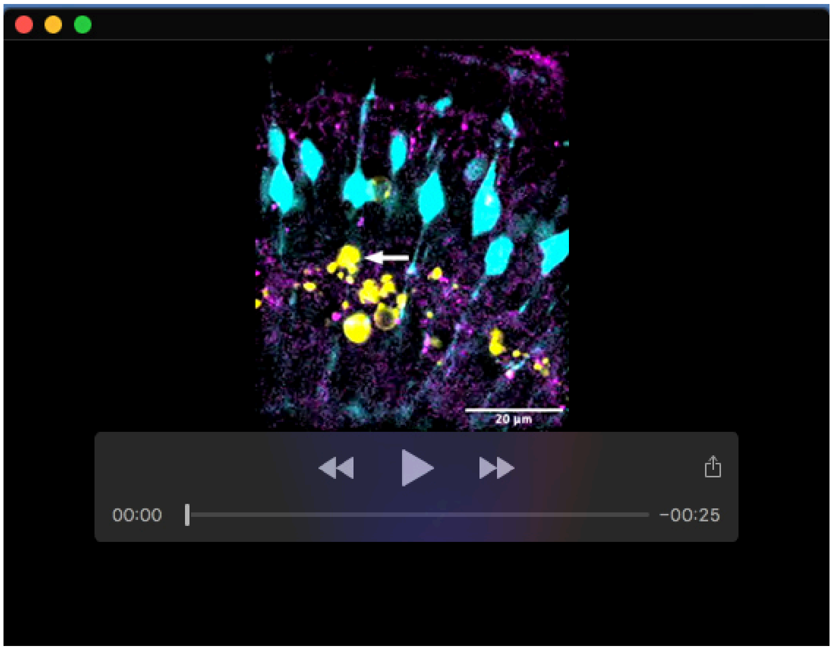

Movie 8. Müller cell phagocytosing an apoptotic cell including lysosomal fusion following engulfment. Müller cell (turquoise) performs phagocytosis of an adjacent dying cell (yellow cell; white arrow). The trafficking of the YFP+ body is coupled with fusion of the phagosome to a lysosome (magenta, white when YFP-lysosomal marker colocalize) which coincides with the diminishing signal from the acid-sensitive YFP cell reporter. Pertains to Figure 4B.

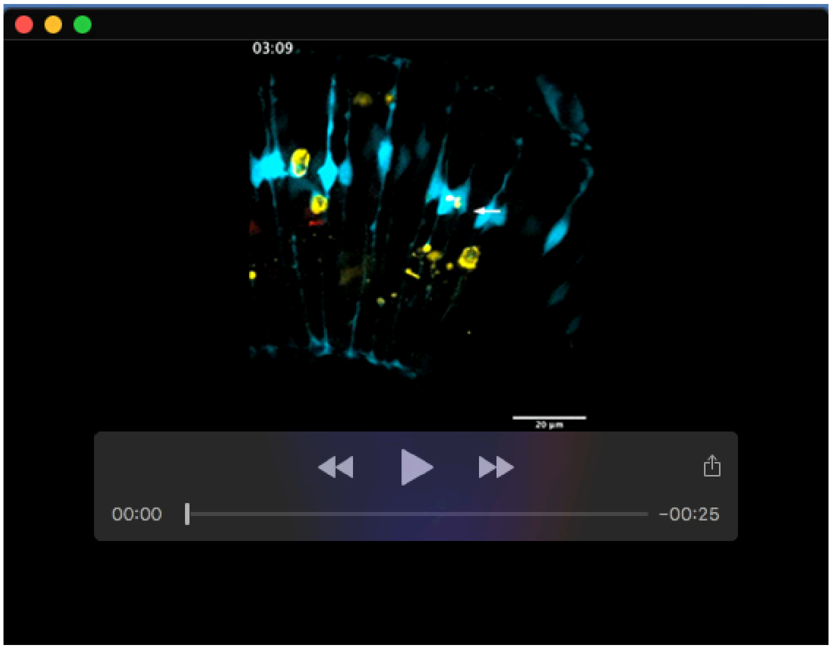

Movie 9. Müller cell partitioning an apoptotic cell body amongst neighboring Müller cells. Upon an initial engulfment of a dying cell (yellow cell; white arrow), the Müller cell (turquoise) fragments the cell body into smaller particles which translocate to neighboring Müller cells. Pertains to Figure 6A.

Development • Supplementary information

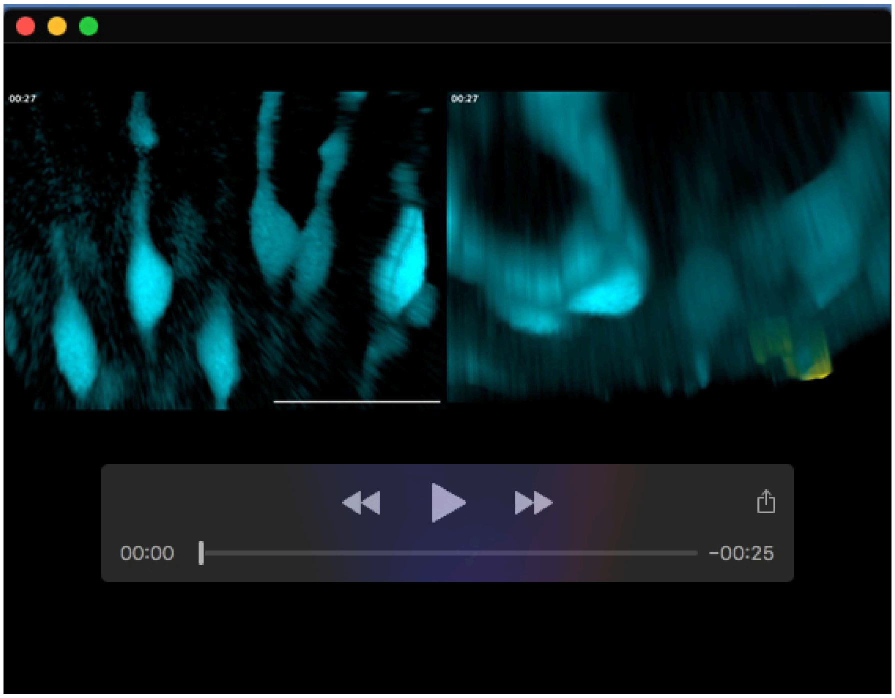

Movie 10. 3D rendering of timelapse presented in Movie 9 (Müller cell partitioning an apoptotic cell body amongst neighboring Müller cells). Left: Front view; Right: Top-Down view. A Müller cell (turquoise) initially enwraps a dying cell (yellow, white arrow) which is then fragmented into smaller portions that translocate into neighboring Müller cells (arrows follow partitioning). Scale bar (left) = 20 µm. Pertains to Figure 6A and Movie 9.

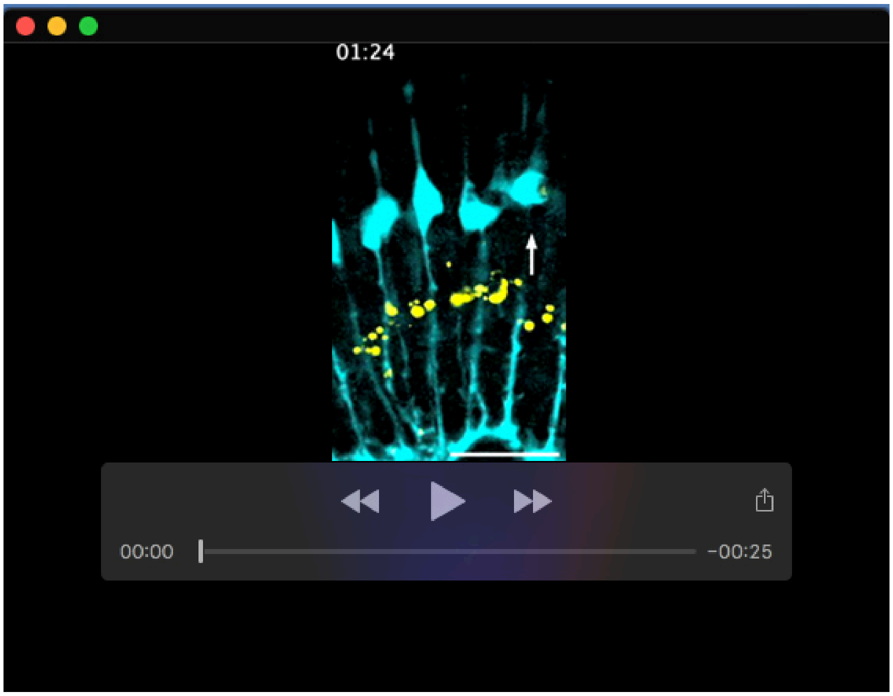

Movie 11. Intercellular pulling for dying cell target. A Müller cell (turquoise) extends a process towards an apoptotic cell target (yellow), and the target appears to be dually engaged by a neighboring Müller cell and the cell that extended the initial process. Retraction of the process shows splitting of the target as the cells end up with smaller pieces of the dying cell. Scale bar = 20 µm. Pertains to Figure 6B.

Development • Supplementary information

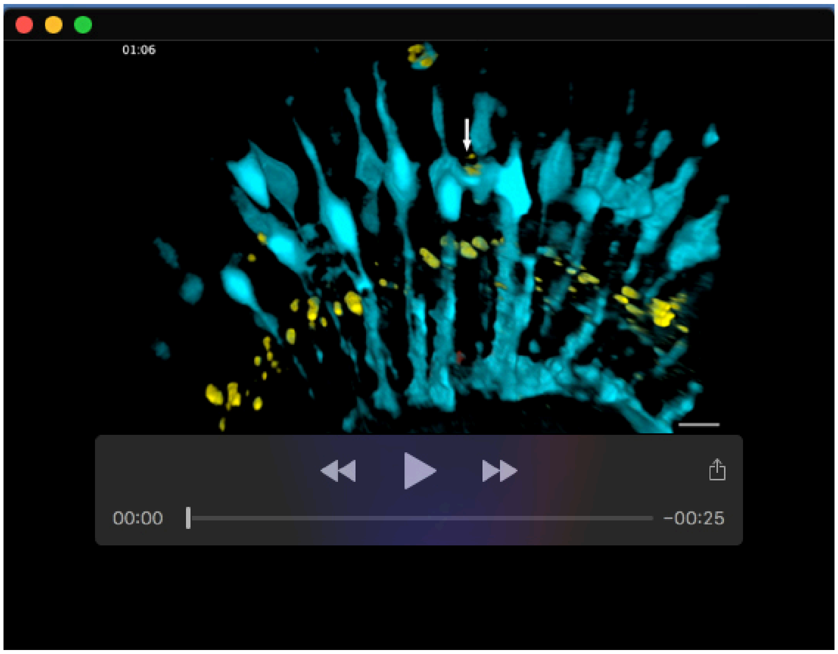

Movie 12. Passing of dying cell between Müller cells. 3-dimensional projection of z-stacks shows the detection of an apoptotic cell (yellow cell; white arrow) being met by the extensions of two Müller cells (turquoise), which appear to loft the cell apically before it is laterally translocated by one Müller cell. Another Müller cell engages the cargo bearing Müller cell and the apoptotic cell body is temporarily shifted towards the next cell, but it is eventually retained and engulfed. In the process, Müller cell bodies frequently extend towards one another and appear to engage in contact. Scale bar = 20 µm. Pertains to Figure 6C.

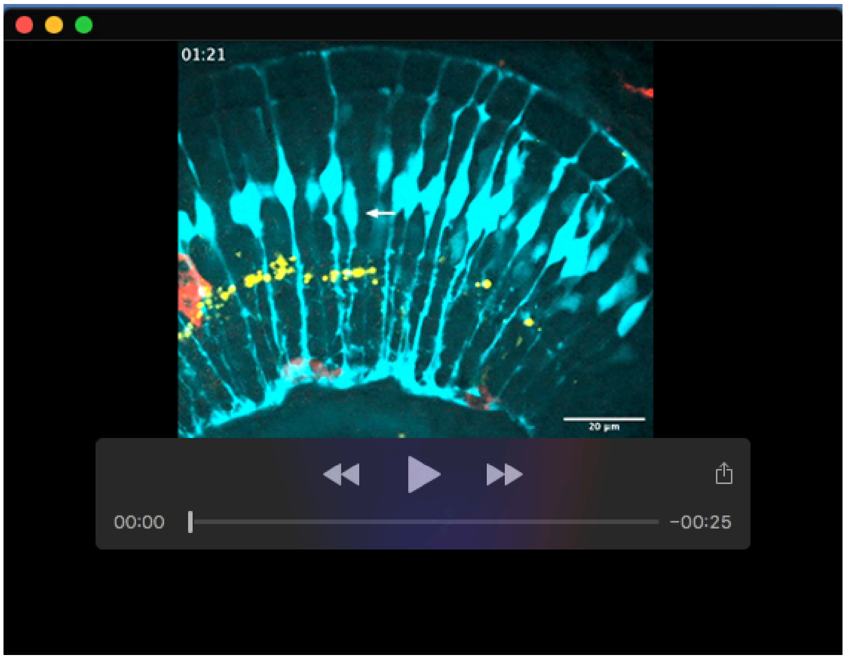

Movie 13. Long cell process extension from Müller cell. Following an engagement with a microglia cell (red), a long process is emitted from a Müller cell (turquoise) that follows a winding path to and from other cells before returning to its source. Pertains to Figure 6D.

Development • Supplementary information

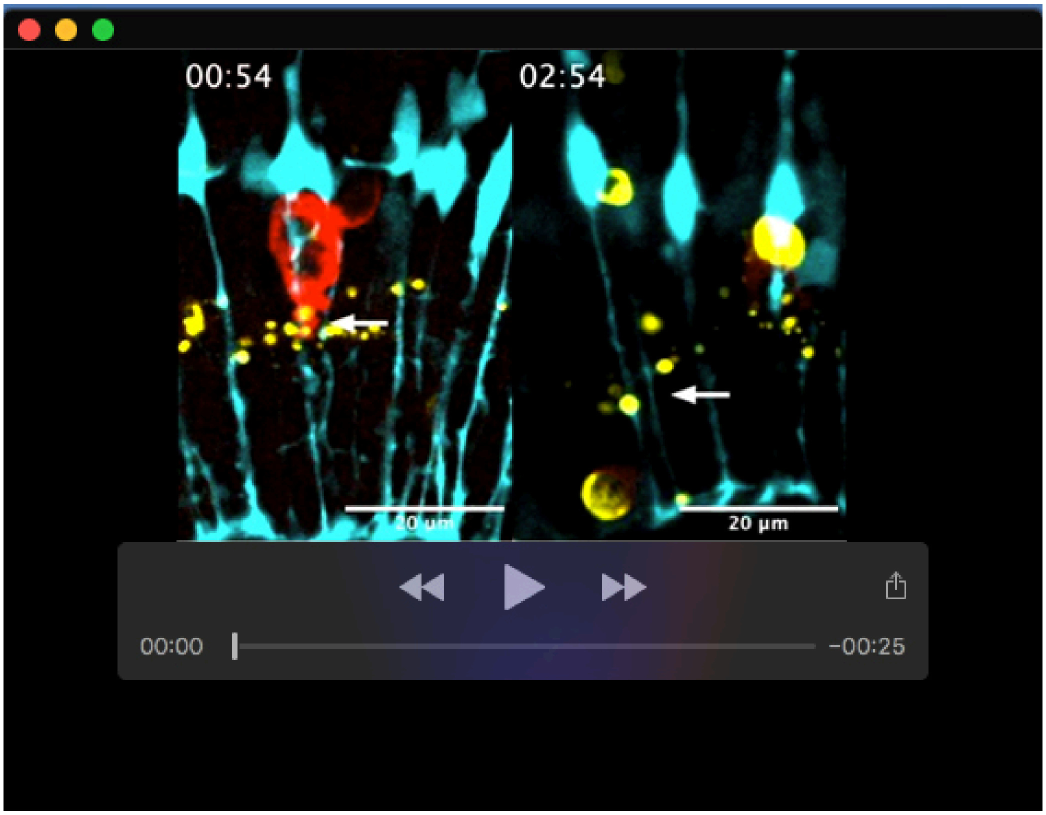

Movie 14. Left: Microglia (red) clearing YFP+ puncta (yellow) in the presumptive inner plexiform synaptic layer through the extension of long processes from the microglia. Not all YFP+ puncta in the vicinity are cleared. Selected engulfment targets are denoted with white arrows. Right: Müller glia (turquoise) clearing YFP+ puncta (yellow) in the presumptive inner plexiform synaptic layer through the extension of small cellular processes that traffic the puncta into the cell (white arrow). Pertains to Figure 7.
